# HOW MANY MOLECULAR MECHANISMS MUST BE ALTERED SIMULTANEOUSLY TO SLOW AGING AND EXTEND LIFESPAN?

**DOI:** 10.1093/geroni/igz038.754

**Published:** 2019-11-08

**Authors:** Nicholas Stroustrup

**Affiliations:** Centre for Genomic Regulation, Barcelona, Spain

## Abstract

Most anti-aging therapeutics are designed to target single molecules, single molecular mechanisms, or single cell types. Yet, to produce a substantial lifespan extension, these interventions would need to act promiscuously and delay the onset of many or all causes of death. In invertebrate models, several molecular-level interventions are known to act this way, yet their downstream action on multiple causes of death remains poorly understood. Recently, we identified a strong mathematical constraint in the way that many different interventions in aging alter all-cause mortality in the nematode Caenorhabditis elegans. Interventions including suppression of IGF/insulin signaling, disruption of the hsf-1 heat shock factor and the hif-1 hypoxia-inducible factor, as well as changes in diet and body temperature, all produce a temporal scaling of lifespan. This temporal scaling suggests a common physiologic path through which diverse, evolutionary-conserved, molecular mechanisms can simultaneously influence all causes of death.

